# Label-free Quantitative Analysis of Protein Expression Alterations in miR-26a-Knockout HeLa Cells using SWATH-MS Technology

**DOI:** 10.1038/s41598-018-34904-8

**Published:** 2019-02-04

**Authors:** Hexiao Shen, Li Li, Zhaowei Teng, Tianqing Meng, Xiangbin Kong, Yan Hu, Yun Zhu, Lixin Ma

**Affiliations:** 1 Hubei Collaborative Innovation Center for Green Transformation of Bio-resources, Hubei Key Laboratory of Industrial Biotechnology,school of Life Sciences, Hubei University, Wuhan, 430062 China; 2 Genecreate Biological Engineering Co., Ltd., National Bio-industry Base, Wuhan, 430071 Hubei China; 3 Department of Orthopedic Surgery, The People’s Hospital of Yuxi City, The 6th Affiliated Hospital of Kunming Medical University, 21 Nieer Road, Yuxi, 653100 Yunan China; 4 Center for Reproductive Medicine, Tongji Medical College, Huazhong University of Science and Technology, Wuhan, 430030 Hubei China; 5 Center for Reproductive Medicine, The First Affiliated Hospital, Wenzhou Medical University, Wenzhou, 325000 China; 6 Department of Gynecology, The Tumor Hospital of Yunnan Province, The 3th Affiliated Hospital of Kunming Medical University, Kunzhou Road, Kunming, 650106 Yunan China; 7 Department of Nephrology, Health Screening Center, The People’s Hospital of Yuxi City, The 6th Affiliated Hospital of Kunming Medical University, 21 Nieer Road, Yuxi, 653100 Yunan China

## Abstract

MicroRNAs (miRNAs) bind to the 3ʹ-untranslated region of target mRNAs in a sequence-specific manner and subsequently repress gene translation. Human miR-26a has been studied extensively, but the target transcripts are far from complete. We first employed the CRISPR-Cas9 system to generate an miR-26a-knockout line in human cervical cancer HeLa cells. The miR26a-knockout line showed increased cell growth and altered proliferation. Proteomics technology of sequential window acquisition of all theoretical mass spectra (SWATH-MS) was utilized to compare the protein abundance between the wild-type and the knockout lines, with an attempt to identify transcripts whose translation was influenced by miR-26a. Functional classification of the proteins with significant changes revealed their function in stress response, proliferation, localization, development, signaling, etc. Several proteins in the cell cycle/proliferation signaling pathway were chosen to be validated by western blot and parallel reaction monitoring (PRM). The satisfactory consistency among the three approaches indicated the reliability of the SWATH-MS quantification. Among the computationally predicted targets, a subset of the targets was directly regulated by miR-26a, as demonstrated by luciferase assays and Western blotting. This study creates an inventory of miR-26a-targeted transcripts in HeLa cells and provides fundamental knowledge to further explore the functions of miR-26a in human cancer.

## Introduction

MicroRNAs (miRNAs) are a subgroup of small RNAs with an average length of 22 nucleotides^1^. miRNAs were first discovered in animals but are now known to exist in plants, fungi, and viruses^1–6^. As non-coding RNAs, miRNAs regulate gene expression at the translational level through sequence-specific binding to the 3ʹ-untranslated region (UTR) of target mRNAs and subsequently repress gene translation^7^. The sequence-specific binding between miRNA and its target mRNA has relatively low stringency requirements; therefore, each miRNA has numerous target mRNAs. Additionally, the existence of multiple recognition sites within the 3ʹ-UTR region of the target mRNA increases the complexity of the miRNA/mRNA interactions^8^. Thus, great effort has been devoted to identify miRNAs and their targets as well as to explore the mechanisms and functions of miRNA regulation of gene expression. It is well known that miRNAs play pivotal regulatory roles in a multitude of cellular processes, including human cancer^9^.

Human miR-26a has been studied extensively, and its multiple functions have been revealed^10^. For example, miR-26a, together with miR-26b and miR-29b, has been found to accelerate osteogenic differentiation of unrestricted somatic stem cells from human cord blood^11^. In addition, Huse *et al*. have reported that miR-26a is regulated by a phosphatase and tensin homolog and that miR-26a amplification facilitates gliomagenesis *in vivo*^8^. miR-26a is also known to function in skeletal muscle differentiation and regeneration^12^ as well as innate immune signaling during *Mycobacterium tuberculosis* infection^13^. However, the downstream target transcripts of miR-26a are far from completely known, and the regulatory mechanisms of miR-26a are complicated, deserving further investigation^10^.

In order to screen for miR-26a target transcripts in a high-throughput assay, we first employed the CRISPR-Cas9 gene editing method to generate an miR-26a-knockout cell line in human cervical cancer HeLa cells. Established in 1951, HeLa, the first continuous human cancer cell line, has been a primary model system for cancer research^14^. HeLa cells also have been employed to study miRNA functions. For instance, miR-21 has been found to promote cell proliferation and repress programmed cell death 4 in HeLa cells^15^. For generation of an miR-26a-knockout mutant in HeLa cells, we chose the recently developed approach of the RNA-guided CRISPR-Cas9 system. Compared to other genome editing systems, such as zinc-finger nucleases and transcription activator-like effector nucleases, the CRISPR-Cas system has the features of an easier design, high specificity and efficiency, suitability for high-throughput screening or multiple gene editing, and adaptions in various cell types^16^. Particularly, the CRISPR-Cas method has been successfully applied in HeLa cells to generate an NADPH oxidase 4 (NOX4)-knockout line to study NOX4 function^17^.

After generation of the miR-26a-knockout cell line, a cutting-edge proteomics technology was employed to compare the protein abundance between the wild-type and the knockout lines at a global level, with an attempt to identify genes regulated by miR-26a in HeLa cells. Sequential window acquisition of all theoretical mass spectra (SWATH-MS), also called MS/MS^all^, is a recently developed label-free strategy for high-throughput quantitative proteomics. This strategy employs a data-independent acquisition mode on mass spectrometry, thus improving the peptide coverage for better protein identification, compared to the traditional data-dependent acquisition methods^18^. For example, Rosenberger *et al*. have employed SWATH-MS to acquire spectra of more than 10,000 human proteins (50.9% of the human proteome) from a collection of human specimens and further enabled the targeted analyses of these proteins^19^.

The aim of this study was to use the SWATH-MS strategy to quantify and compare proteins in both wild-type and miR-26a-knockout HeLa cells. Functional classification of the proteins with altered expression was performed, and several of the identified proteins were confirmed by western blotting and parallel reaction monitoring (PRM). The results from the three approaches showed satisfactory consistency, indicating the reliability of the quantification results by the SWATH-MS experiments. We also employed bioinformatics tools to predict miR-26a targets and compared the predictions to the proteomics-revealed putative targets. Among the targets that were identified by proteomics and bioinformatics, direct regulation by miR-26a was validated by luciferase assays and Western blotting, including NUF2, CDK6, MYPN, DNAJC9, USP47, and PPA1. This study created an inventory of miR-26a-targeted genes in HeLa cells and provides fundamental knowledge to further explore the functions of miR-26a in human cancer.

## Results

### Generation of the miR-26a-knockout HeLa cell line with the CRISPR-Cas9 gene editing system

**Figure 1:**
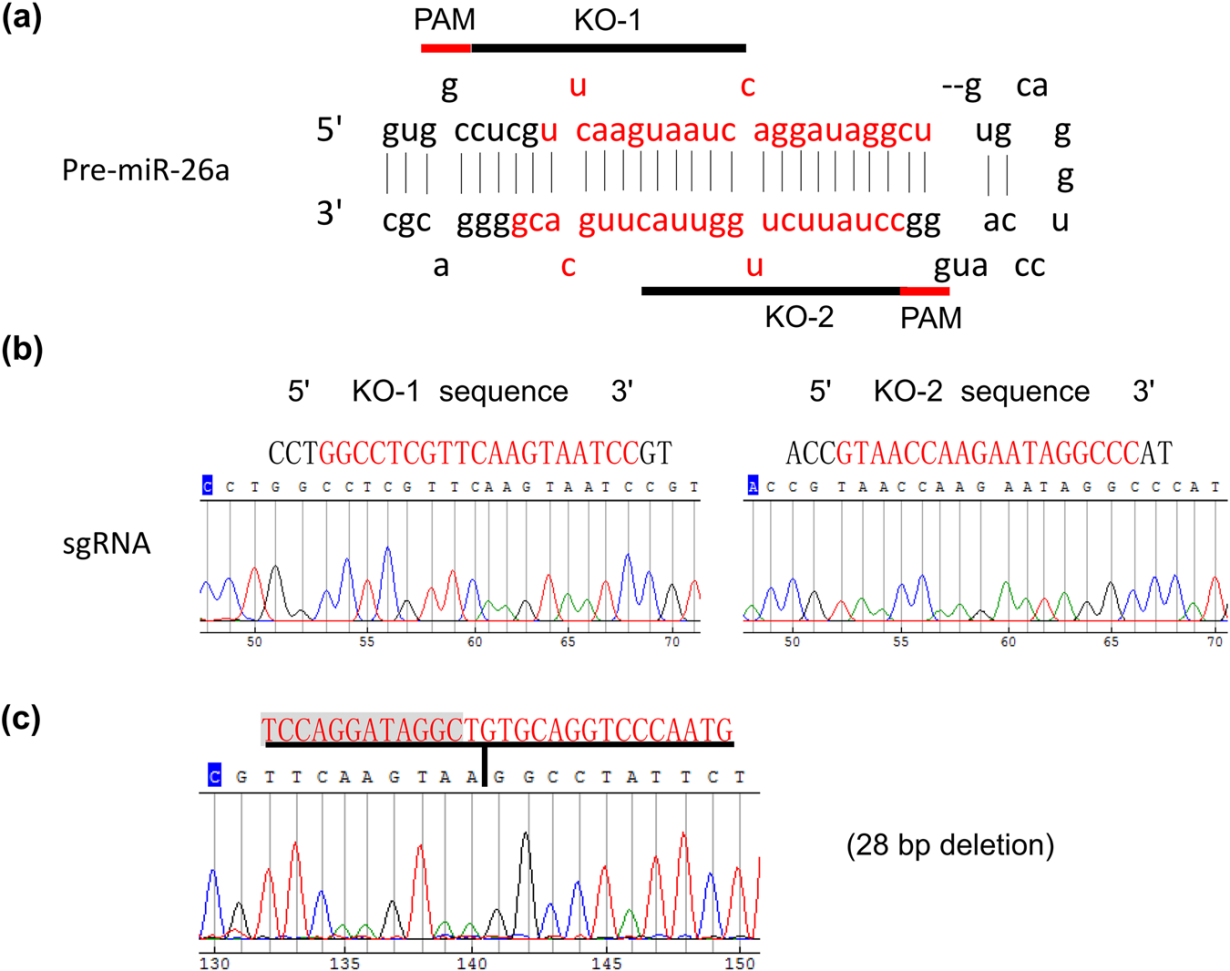
Generation of the miR-26a-knockout line in HeLa cells using the CRISPR-Cas9 system. (**a**) Two sgRNAs were designed for miR-26a depletion by CRISPR DESIGN (http://crispr.mit.edu/). (**b**) The sequencing results showed that the two sgRNAs were appropriately inserted into the lentiCRISPRv2 vector. (**c**) The sequencing data revealed that a 28-bp deletion in the target site was introduced by the CRISPR-Cas9 system.

We generated the miR-26a-knockout HeLa cell line using the CRISPR-Cas9 gene editing system. Two single-guide RNAs (sgRNAs) were designed for miR-26a depletion (Fig. 1a) and cloned into the lentiCRISPRv2 vector. Insertion into the vector was confirmed by DNA sequencing (Fig. 1b). After transfection, a mutation caused by a 28-bp deletion in the targeted site was also confirmed by nucleotide sequencing (Fig. 1c). The miR-26a level in the knockout line was significantly lower (76% decrease) than that in the wild-type cells (Supplemental Fig. S1).

### miR-26a-deficient cells exhibit increased cell growth and proliferation

**Figure 2:**
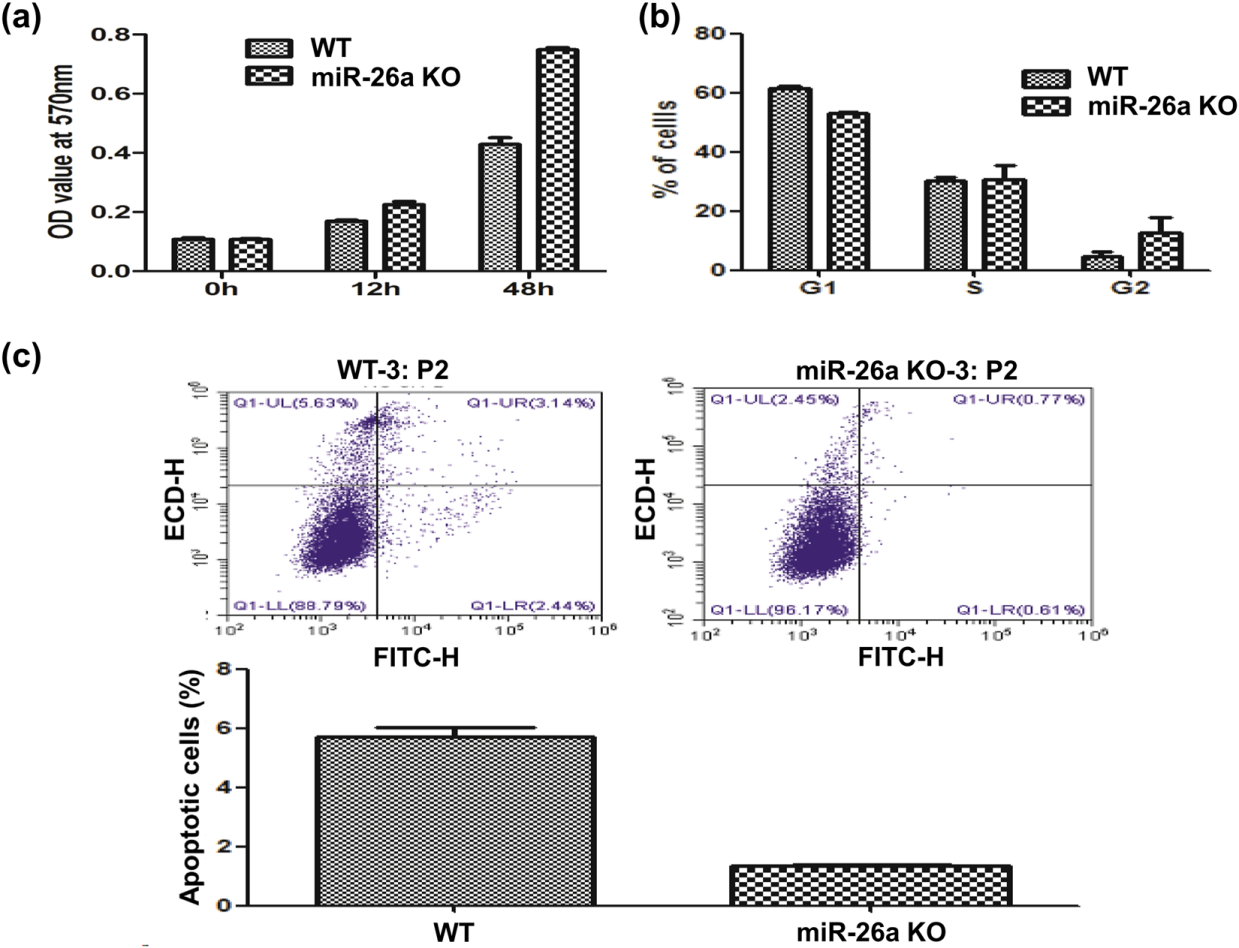
The miR-26a-deficient cells exhibited an altered growth rate and cell cycle. (**a**) The miR-26a-knockout HeLa cells had a significantly increased cell growth rate, compared to the wild-type cells. (**b**) The effect of miR-26a knockout on cell cycle progression. The percentage of cells in the G2 phase was significantly increased after miR-26a knockout. (**c**) Apoptosis was determined by annexin V staining and flow cytometry.

Compared to the wild-type HeLa cells, the miR-26a-deficient cells (knockout line) exhibited an increased cell growth (Fig. 2a). Therefore, we also compared the proliferation of the wild-type and the knockout lines. A much higher percentage of cells staying in the G2 phase was observed in the miR-26a-deficient cells (Fig. 2b). We also determined the extent of apoptosis by annexin V staining and flow cytometry and found that the percentage of apoptotic cells in the miR-26a-deficient line was significantly less than that in the wild-type HeLa cells (Fig. 2c). These data suggested that miR26-a targets the functions of cell growth and proliferation. This conclusion is also supported by previous reports^20,21^.

### Comparative proteomics between the wild-type and the miR-26a-knockout line*s* using SWATH-MS

First, we designed the experimental flowchart for SWATH-MS-based quantitative proteomics (Supplemental Fig. S2). Three biological replicates of both wild-type (NC-HeLa) and miR-26a-knockout (KO-HeLa) lines were prepared, with each containing 10^7^ cells. Proteins were extracted from the harvested cells and quantified. A pooled sample of the above-mentioned six biological replicates (three for NC-HeLa cells and three for KO-HeLa cells) were used to generate a reference library. The six individual samples were analyzed with SWATH 2.0 software (Sciex, USA). All of the acquired data were used for statistical analysis and relative protein quantitation across all six samples in both groups. Proteins with significantly altered expression levels were further analyzed to reveal potential miR26-a functions, and some were selected for validation using PRM and western blotting.

**Figure 3:**
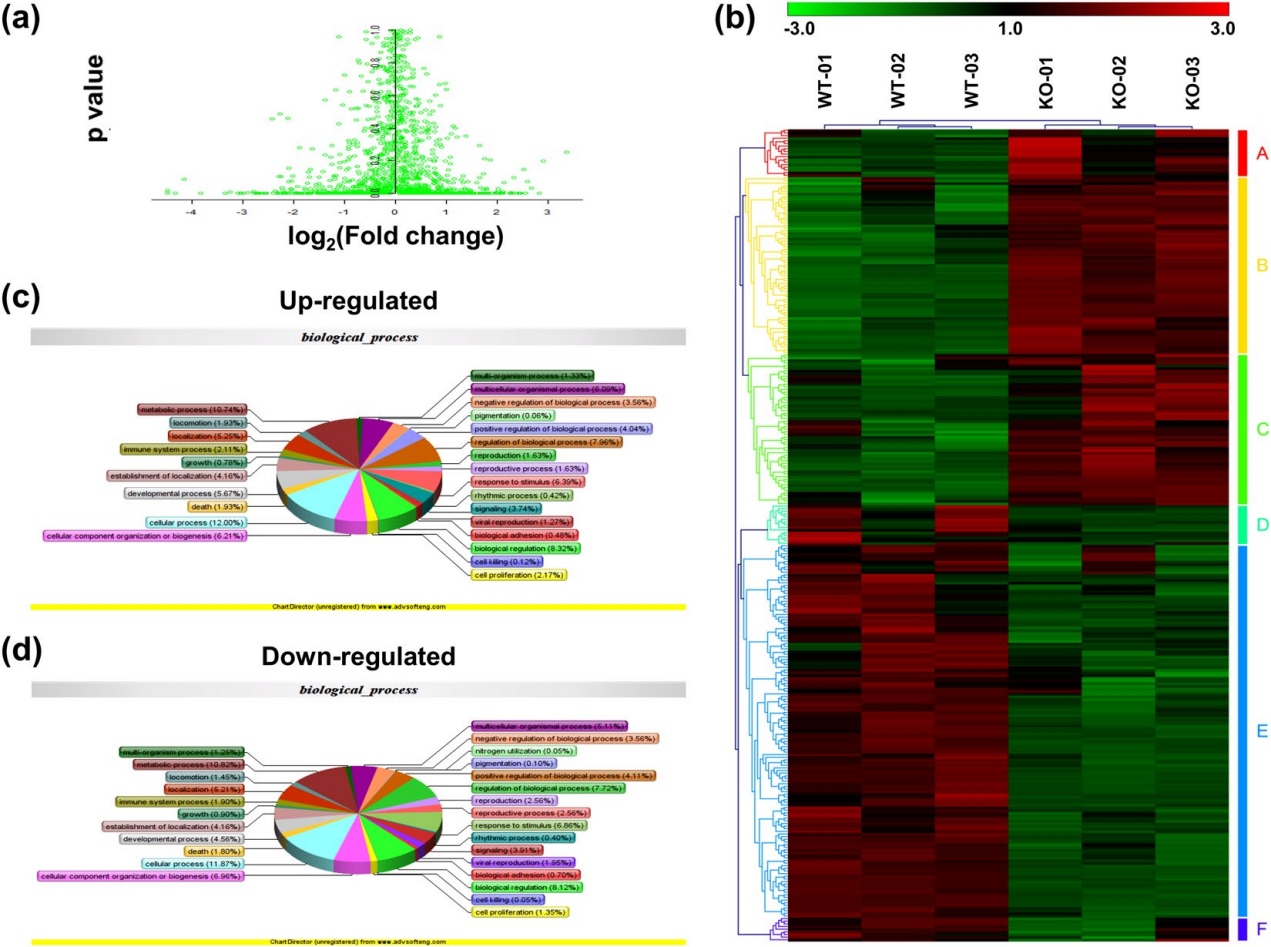
SWATH-MS analysis revealed differentially expressed proteins between the wild-type and knockout cell lines. (**a**) Volcano plot of the p values and fold changes (log2) of the 1646 relatively quantified proteins from the two groups. (**b**) Heat map analysis of the 466 proteins with significantly altered expression levels. The heat map was produced via Genesis^78^. All of the significantly altered proteins were grouped into six clusters (labeled as A–F on the right side of the heat map) based on the protein expression patterns. (**c**) GO analysis of the upregulated proteins in the miR-26a-knockout line, compared to the wild-type line. (**d**) GO analysis of the downregulated proteins in the miR-26a-knockout line, compared to the wild-type line.

A total of 3201 proteins were identified in the wild-type and miR-26a-knockout HeLa cell lines, and 1646 proteins from the two groups of samples were relatively quantified (Supplemental Fig. S3A and Supplemental Table 1). We also found that approximately 68.31% of the proteins had quantitative coefficients of variation (CVs) < 25% within the biological replicates of the miR-26a-knockout group, and approximately 66.33% of the proteins had quantitative CVs <25% within the biological replicates of the wild-type group (Supplemental Fig. S3B). The Pearson correlation coefficients of the protein abundance across the three biological replicates within each group were all over 0.9 (Supplemental Fig. S4). These analyses indicate a good reproducibility of the biological replicates in each group. Based on a threshold of *p* < 0.05 (Student’s *t* test between the wild-type and knockout lines) and a fold change >1.5, 466 proteins were found to have significantly altered expression levels (Supplemental Fig. S3), with 214 proteins upregulated and 252 proteins downregulated in the miR-26a-knockout line compared to the wild-type line. Figure 3a shows the volcano plot of the p values and fold changes (log2) of the 1646 relatively quantified proteins from the two groups. In addition, Fig. 3b shows the heat map analysis of the 466 proteins with significantly altered expression levels.

Functional classification of these proteins revealed their functions in stress response, proliferation, localization, development, signaling, etc. (Fig. 3c,d). For example, a large portion (106/214) of the miR-26a-repressed proteins (i.e., upregulated proteins in the knockout line) are responsive to stimuli (Supplemental Table 3). Among these proteins, the putative heat shock protein (HSP) 90 showed a 76.4% increase in expression in the miR-26a-knockout line, compared to the wild-type cells. HSPs have been reported to be induced in a wide range of human cancers^22^, and the high levels might provide an environment conducive for cancer development^23^. Specifically, inhibition of HSP90 results in degradation of oncogenic proteins in cancer cells; thus, HSP90 has been proposed as a promising target for cancer chemotherapeutics^24^. In addition, HSP90 has been found to be a target of miR-223, miR-27a, and miR-499^25–27^. Because HSPs are known to regulate polypeptide binding, folding, and release through ATP binding and hydrolysis^23^, it is not surprising that we observed that a couple of ATPases were upregulated in the absence of miR-26a, including mitochondrial ATP synthase subunit delta and Na^+^/K^+^-transporting ATPase subunit beta-3.

Another distinguished group includes proteins involved in signaling pathways (Supplemental Table 3). The guanine nucleotide-binding protein (G-protein)-related signaling pathway functions in a variety of cellular processes, e.g., metabolism, development, and reproduction. In addition, G-protein-coupled receptors (GPCRs) are a large family of signal-conveying membrane proteins. Upon ligand binding to GPCRs, the GPCR conformational change facilitates activation of the heterotrimeric G protein and subsequent related signaling pathways^28^. GCPRs are newly emerged potential targets for cancer treatment^29^. The G-protein alpha subunit has been reported to be a potent tumor suppressor in childhood brain tumors^30^. Here, the G-protein subunit alpha-13 and the signal recognition particle receptor subunit alpha, two proteins involved in the G-protein signaling pathway, were found to be repressed by miR-26a. Such a linkage between G-protein signaling components and miR-26a has been rarely reported previously.

The 14-3-3 proteins, with specific binding activity to phosphorylated serine and threonine, are multifunctional in various biological processes, such as cell proliferation and migration, cancer progression, etc.^31^. These proteins have been reported to be regulated by miR-152 and miR-451^32,33^. Here, we observed that 14-3-3 sigma was repressed by miR-26a, expanding the regulatory interactions between miRNA and 14-3-3 family proteins. Protein phosphorylation and dephosphorylation are universal key mechanisms for cellular signal transduction. Not surprisingly, several protein phosphatases and kinases have been found to be targets of miR-26a, including serine/threonine-protein phosphatase 2 A (PP2A), protein kinase C (PKC, alpha type), serine/threonine-protein kinase 26, mitogen-activated protein kinase 1 (MAPK1), and cyclin-dependent kinases. PP2A as well as PKC have been proven to be critically involved in the control of cellular growth, cell survival, and cancer progression^34,35^. In addition, MAPK1 has recently been reported to be a target of miR-329-3p and function as a suppressor of cell proliferation, migration, and invasion in cervical cancer^36^. These observations indicate that not only post-translational modification but also miRNA-mediated regulation at the translational level are critical in oncogenesis.

Another group of upregulated proteins is involved in the ubiquitin-proteasome system, a key regulator of cell growth and apoptosis, including proteasome subunit alpha type, proteasome assembly chaperone 3, and several E3 ubiquitin-protein ligases and ubiquitin-conjugating enzymes. This observation suggests an enhancement in the intracellular protein degradation of the faster-growing knockout cell line. Several pieces of evidence have shown that proteasome inhibitors induce apoptosis and thus are cytotoxic to cancer cells, with the potential to be applied to target proteasomes in cancer therapy^37,38^. Moreover, it has been reported that miR-101 targets the proteasome assembly factor POMP and suppresses tumor cell proliferation^39^. The mechanism of how miR-26a, with these identified putative targets in the ubiquitin-proteasome system, acts as an anticancer miRNA deserves further investigation.

Additionally, several proteins function in cell growth, death, and proliferation (Supplemental Table 3), including cyclin-dependent kinases (CDKs) 1, 4, and 6. These CDKs were found to be regulated by miR-26a, and these kinases are known to be key regulators of the cell cycle^40,41^. Other groups of miR-26a-repressed proteins included vesicle coat proteins (coatomer subunits beta, gamma, and delta), translation initiation factors (eukaryotic translation initiation factors 2, 3, and 5), and non-motor actin binding proteins (shootin-1, calponin-3, destrin, tropomodulin-3, fascin, etc.). A subunit of coatomer protein has been reported to harbor miR-152 (encoded by the first intron of the gene), which shows tumor suppressive activities *in vitro* and *in vivo*^42^. Furthermore, some miRNAs are known to regulate cytoskeletal organization, e.g., miR-143, miR-145, and miR-940^43,44^.

Our study identified 252 miR-26a-induced proteins (i.e., downregulated proteins in the knockout line; Supplemental Table 2). These proteins are mainly involved in stress responses, development, signal transduction, localization establishment, and reproduction (Supplemental Table 3; Fig. 3d). Moreover, redox regulation is a typical phenomenon linked to stress responses, in which reactive oxygen species (ROS) production is promoted upon stress as either a consequence or a signaling event, especially in cancer^45,46^. Proteins involved in redox regulation were found to be decreased in the miR-26a-deficient line, including glutathione peroxidase, mitochondrial glutathione reductase, and thioredoxin-dependent peroxide reductase.

Several ribosomal proteins (RPs) were found to be downregulated in the miR-26a-knockout cell line, including the 60 S ribosomal proteins L13, L18, L16, L30, and 40 S as well as the ribosomal proteins S10, S13, S18, and S24. RPs are ribosomal components that function in ribosome biogenesis and protein translation. Amsterdam *et al*.^47^ have reported several RPs as cancer genes in zebrafish and have proposed the roles of RPs in human tumorigenesis. In addition, Guimaraes and Zavolan have observed consistent dysregulation of individual RP expression in a range of cancer types^48^. On the other hand, RP gene haploinsufficiency has been found to be a common vulnerability of human cancers^49^. This observation reasonably explains our finding of downregulation of some RPs in the miR-26a-deficient line, which showed tumorous features.

Proteins showing a decreased abundance in the miR-26a-knockout line also included the category of cellular localization and transport, e.g., charged multivesicular body protein 5, clathrin heavy chain 1, general vesicular transport factor p115, importin subunit alpha-7, and nuclear pore membrane glycoprotein 210. Vesicular transport and signaling pathways are known to play important roles in tumorigenesis; and dysregulated vesicle trafficking systems have been observed in a variety of cancer cells^50^. The small GTPase Rab31, a regulatory factor of the vesicular transport system, also was found to be decreased in the knockout line. Elevation of the Rab31 level has been observed in breast cancer^51^. Our results suggest that the regulatory role of miR-26a on the vesicular transport system is linked to oncogenesis. Altogether, our proteomics analysis created an inventory of miR-26a-regulated transcripts in HeLa cells and provides fundamental knowledge to further explore the functions of miR-26a in human cancer.

### Pathway analysis of miR-26-targeted proteins

**Figure 4:**
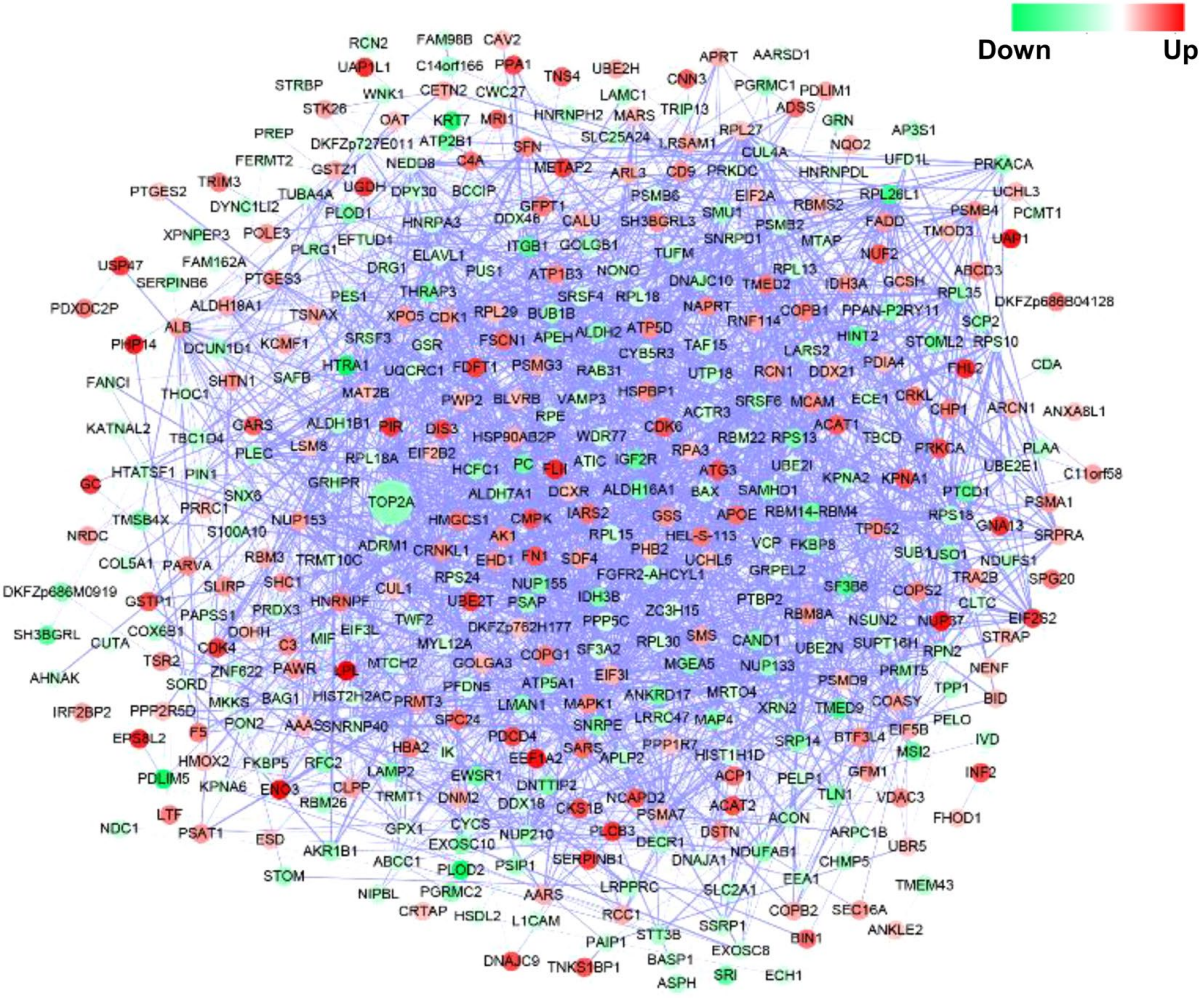
Pathway analysis of proteins regulated by miR-26a as identified by the proteomics analysis. A total of 1950 interactions were identified among the 413 proteins (see Supplemental Table 2 for protein name abbreviations). The circle colors (see color scale for reference) indicate the fold change of the proteins revealed by SWATH-MS; the thickness of the line between two proteins represents the reliability of the interaction.

Next, we performed pathway analysis of the proteins with significantly altered expression levels (Fig. 4). A total of 1950 interactions were identified among the 413 proteins (see Supplemental Table 2 for protein name abbreviations). DNA topoisomerase 2-alpha (TOP2A) showed interactions with 63 other proteins, representing the most distinguished protein node in the interaction network. *TOP2A* has been found to be amplified in breast and some other cancers^52^. Cowell *et al*.^53^ also have reported an interaction between TOP2A and P53, a crucial tumor suppressor^54^. The multiple interactions with TOP2A and other miR-26a-regulated proteins suggest that the TOP2A-related pathways play a critical role in carcinogenesis and/or anticarcinogenesis.

### Validation of some proteins of interest using western blot and PRM

**Figure 5:**
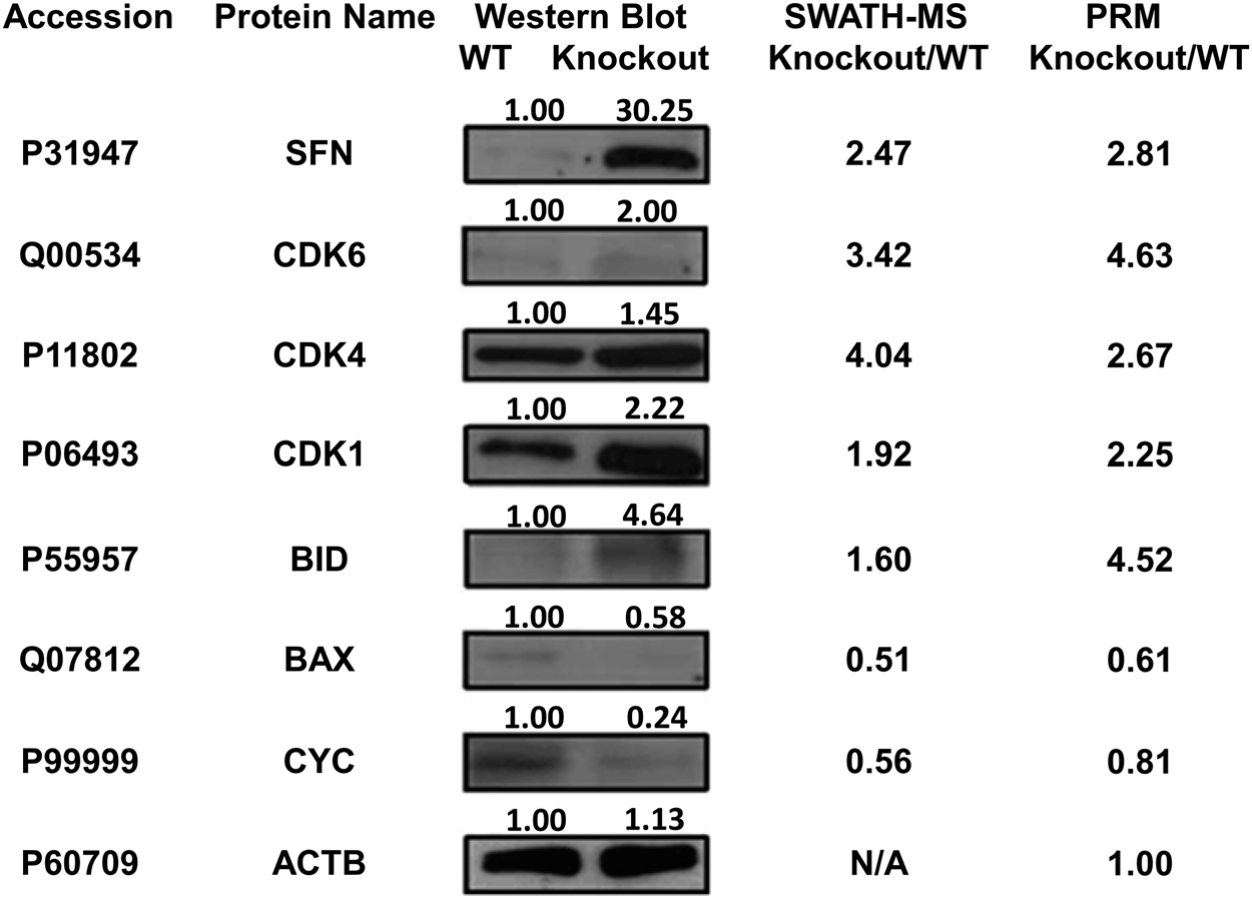
Relative quantitation of miR-26a-regulated proteins that were involved in the p53 signaling pathway using sequential window acquisition of all theoretical mass spectra (SWATH-MS), parallel reaction monitoring (PRM), and western blot. The SWATH-MS and PRM results are presented as ratios (mean ± standard error). Full-length western blots are presented in Supplemental Fig. S4.

Together with the observation that the miR-26a-knockout line showed increased cell growth and altered proliferation, we chose several proteins involved in cell proliferation to validate the quantification results by western blot and PRM. All the proteins we tested showed consistent trends of abundantly altered expression levels (Figs 5 and S4). CDK1, CDK4, and CDK6 were found to be upregulated in the miR-26a-knockout line, suggesting their suppression by miR-26a. CDK1 is a central regulator that promotes mitosis; while CDK4 and CDK6 are activated in the early G1 stage to block retinoblastoma repression^40,41^. The 14-3-3 protein (*SFN*) and BH3-interacting domain death agonist p15 (*BID*) are also potential miR-26a-suppressed genes. Cytochrome c (CYC) and the apoptosis regulator BAX were downregulated in the miR-26a-knockout line, suggesting their activation by miR-26a. All these proteins showed the same trend of abundantly altered expression levels in the three experimental approaches, i.e., SWATH-MS, PRM, and western blot (Fig. 5). The satisfactory consistency of the results from both quantitative (SWATH-MS and PRM) and semi-quantitative (western blot) methods indicates the reliability of the quantification results by the SWATH-MS experiment.

### Sequence analysis of candidate targets identified from SWATH proteomics

**Figure 6:**
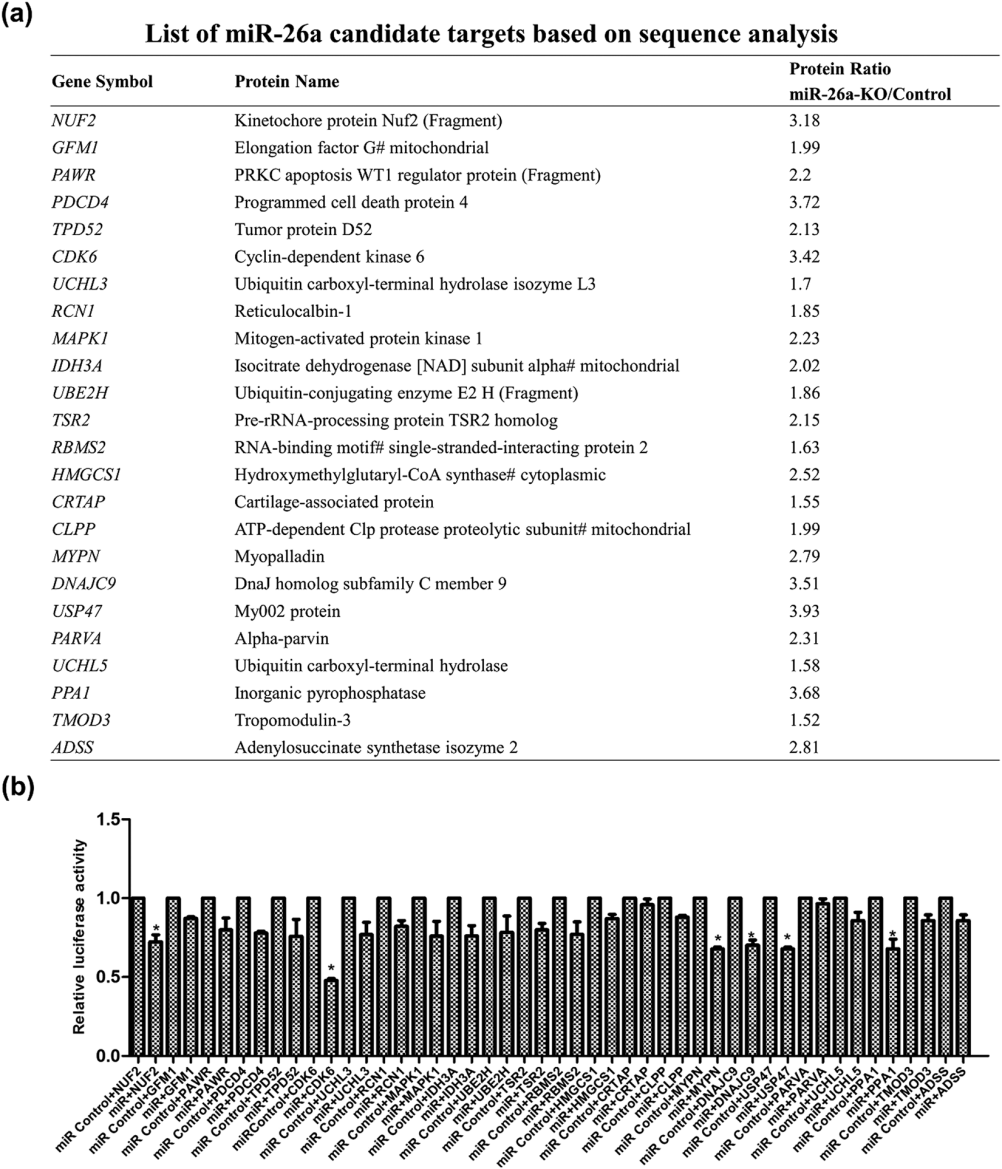
Sequence analysis of candidate targets identified from SWATH proteomics. A partial list of candidate targets of miR-26a identified by proteomics (**a**) and verification of miR-26a targets by luciferase assays (**b**). The transcripts of all genes identified by proteomics were searched against nucleotide sequence databases to obtain their 3ʹ-UTR sequences. Several computational programs were employed to predict potential targets of miR-26a, which were then compared with our proteomic data. Luciferase assays were performed to verify miR-26a candidate targets identified by our proteomics. Data are relative luciferase activities normalized to the corresponding transfections with control oligo and shown as the mean ± SD of five replicates. Asterisks indicate *p* < 0.05 using the two-tailed t-test (miR-26a mimic vs. control oligo).

In order to find miR-26a seed matches in transcripts of candidate targets identified from SWATH proteomics, the transcripts of all genes identified by proteomics were searched against nucleotide sequence databases to obtain their 3ʹ-UTR sequences. Several programs (i.e., PicTar, TargetScan, miRanda, and RNA22) were employed to predict potential targets of miR-26a, which were then compared with our proteomic data. Figure 6a lists the putative targets identified by SWATH analysis (with a higher level in the knockout line) that were predicted as miR-26a targets by these three prediction methods.

### Verification of miR-26a targets by luciferase assays

**Figure 7:**
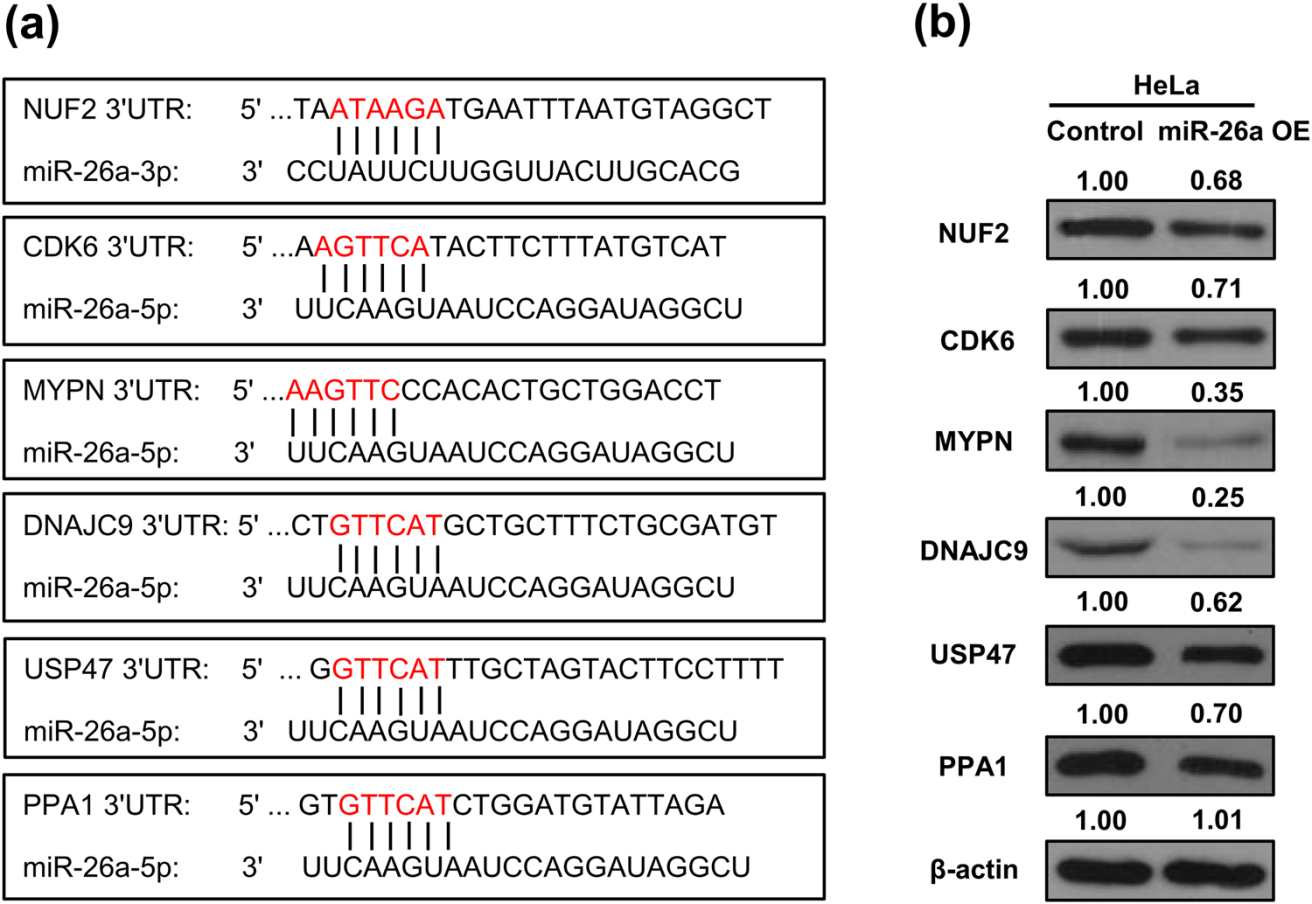
Validation of interaction between miR-26a and six potential targets, i.e., *NUF2*, *CDK6*, *MYPN*, *DNAJC9*, *USP47*, and *PPA1*. (**a**) The six potential targets contain seed sequences in the 3′ UTRs matched to miR-26a, based on the bioinformatics algorithms. (**b**) Western blotting revealed downregulation of the six potential targets in the miR-26a over-expression (OE) line.

We performed luciferase assays to determine if miR-26a directly regulates the putative targets identified from SWATH analysis. Twenty-four candidate targets with seed matches in the 3ʹ-UTR regions (Fig. 6a) were tested. Six out of the 24 candidates, i.e., *NUF2*, *CDK6*, *MYPN*, *DNAJC9*, *USP47*, and *PPA1*, showed significant reduction (*p* < 0.05) as illustrated in Fig. 6b. Particularly, *CDK6*, which showed an induced expression level in the miR-26a-knockout line and was further validated by western blot and PRM (Fig. 5), was found to be directly regulated by miR-26a (Fig. 6b). The six targets, contain seed sequences in the 3′ UTRs matched to miR-26a (Fig. 7a). Additionally, the expression levels of these targets were downregulated in the miR-26a over-expression line, revealed by Western blotting (Fig. 7b).

## Discussion

Elucidation of each miRNA, especially the ones involved in carcinogenesis, and its targets, although challenging, is critical to understand the function of miRNAs and the interaction network. The recently established CRISPR-Cas system has been successfully applied in HeLa cells to generate a knockout line to study gene function^17^. This gene editing system can be easily designed with a high specificity and efficiency. Therefore, we chose the CRISPR-Cas9 gene editing method to generate an miR-26a-knockout cell line in human cervical cancer HeLa cells in order to screen for miR-26a target transcripts in a high-throughput assay. Chang and colleagues have demonstrated the successful application of CRISPR-Cas 9 to knock down miRNAs in human colon cancer cells^55^. Knockout lines of miR-17, miR-200c, and miR-141 were generated, and all showed satisfactory robustness, specificity, and stability. The availability of these miRNA knockout lines in cancer cells provides an invaluable tool to characterize miRNA function and to screen for miRNA targets in order to further understand the regulatory mechanisms of miRNAs in oncogenesis.

We observed that the miR-26a-deficient cells (knockout line) exhibited an increased cell growth and an altered proliferation pattern (Fig. 2). Additionally, the knockout line demonstrated a lower percentage of apoptotic cells, compared to the wild-type HeLa cells, showing a more tumorous phenotype (Fig. 2c). It has been known for many years that miR-26a functions as a tumor suppressor miRNA in breast cancer. Gao *et al*. have reported the downregulation of miR-26a in breast cancer cells and clinical specimens^56^. In addition, miR-26a impacts cell proliferation as well as cancer migration through several carcinogenic processes^56^. Thus, the linkage between miR-26a and carcinogenesis was confirmed by our proteomics study.

SWATH-MS analysis revealed that miR-26a-regulated proteins are linked to carcinogenesis, including some potential cancer therapy targets, e.g., HSP, G-protein subunit, GPCR, proteasomes, etc. Our results also highlight the role of miR-26a on phosphorylation and redox regulation, two essential post-translational modifications functioning in oncogenesis^57,58^. Many kinase-dependent signaling pathways play important roles in the cell cycle, such as MAPK and CDK signaling cascades. Dysregulation of these cascades is common in a variety of cancers^58^. This is also consistent with our observation that MAPK1, CDKs, as well as 14-3-3 and several other kinases were found to have an increased expression in the miR-26a-knockout line. It is widely accepted that miRNAs generally repress gene expression. However, Place *et al*. have reported that miR-373 can induce gene expression via a complementary sequence in the promoter region^59^. In our study, 252 miR-26a-induced proteins were identified, including some proteins involved in redox regulation. The role of antioxidant systems in cancer progression has been controversial for decades. Harris *et al*. have reported that glutathione and thioredoxin antioxidant pathways are essential for tumor initiation and progression, suggesting that the activation of antioxidant systems is an adaptive response in cancer cells to maintain the redox homeostasis in order to restrict ROS elevation during rapid cell growth^45^.

Based on the potential miR-26a targets revealed by SWATH proteomics, bioinformatics approaches and luciferase assays were further employed to validate the interaction between miR-26a and the potential targets. NUF2, CDK6, MYPN, DNAJC9, USP47, and PPA1, all containing matched seeds to miR-26a, were identified as targets by luciferase assays. Additionally, the expression of these six genes were suppressed in the miR-26a overexpression line, suggesting they are bona fide targets of miR-26a. NUF2 has been reported to be associated with several human cancers including lung cancer, colorectal cancer, and prostate cancer^60^. NUF2 is a direct target of miR-3613-3p which participate in cell proliferation and cell cycle in hepatocellular carcinoma. CDK6 has been found as a target of several miRNAs, including miRNA-195, miRNA-29c, miRNA-218, miRNA-504, and miR-6883, and linked to bladder cancer, colon cancer, oral verrucous carcinoma, and medulloblastoma^61–67^. USP47 was found as a target of miRNA-204-5p and involves in gastric cancer cell proliferation^68^. Here we demonstrated NUF2, CDK6, and USP47 as miR-26a targets in HeLa cells. Their function related to cervical cancer deserves further investigation. Additionally, MYPN, DNAJC9, and PPA1 were found as miRNA targets, for the first time.

In conclusion, this study, for the first time, created an inventory of miR-26a-targeted genes in HeLa cells through a large-scale proteomics approach. In addition, the findings from this study provide fundamental knowledge to further explore the functions of miR-26a in human cancer.

## Materials and Methods

### Cell culture

The HeLa cell line was purchased from the Type Culture Collection of the Chinese Academy of Sciences (Shanghai, China) and was maintained in HyClone™ Dulbecco’s modified Eagle medium (DMEM; GE Healthcare Life Sciences, USA) containing 10% fetal bovine serum (FBS) (Gibco^®^, Thermo Fisher Scientific Inc., USA), 2 mM glutamine, 50 U/mL penicillin, and 50 mg/mL streptomycin at 37 °C in a humidified atmosphere with 5% CO_2_.

### Generation of the miR-26a-knockout cell line with the CRISPR-Cas9 system

The lentiCRISPRv2 vector capable of producing high-titer virus^69^ was a gift from Dr. Feng Zhang through Addgene (Addgene plasmid #52961; https://www.addgene.org/crispr/). The sgRNAs targeting miR-26a were designed at CRISPR Design (http://crispr.mit.edu/) to be located right before the protospacer adjacent motif, a DNA sequence immediately following the Cas9-targeted DNA sequence. All the target sequences were amplified and cloned into the lentiCRISPRv2 vector and verified by DNA sequencing. To produce the lentivirus, HEK 293 T cells seeded in 100-mm plates were cotransfected with 4.0 μg of lentiCRISPRv2 plasmids, 3.0 μg of psPAX2 (Addgene plasmid # 12260), and 1.0 μg of pMD2.G plasmids (Addgene plasmid #12259) using polyethyleneimine (Polysciences Inc., USA), according to the manufacturer’s instructions. HEK 293 T cells were grown in DMEM (ATCC, USA) containing 10% FBS for 3 days. The supernatant of the transfected cells containing lentivirus was then harvested, passed through a 0.45-µm filter, and stored at −80 °C. For transduction with lentivirus, the HeLa cells (2 × 10^5^) were seeded in 6-well plates, and the spin-transduction was performed by centrifuging the plate coated with 8 μg/mL polybrene at 2600 rpm and 25 °C for 20 min. The cells were cultured for 12 h, followed by the addition of fresh DMEM supplemented with 10% FBS, and treated for six days with 0.5 μg/mL puromycin for selection. The puromycin-resistant cells were then expanded in regular culture medium.

### miR-26a expression level analysis

Trizol was used to isolate RNA from HeLa cells, and 1 µg of total RNA was transcribed into cDNA, according to the manufacturer’s protocol (High-Capacity cDNA Reverse Transcription Kit, Applied Biosystems, USA). The SYBR PCR system with a 20-µL volume was used to detect miR-26a with the following primers: RT primer, TCAACTGGTGTCGTGGAGTCGGCAATTCAGTTGAGCGTGCAAG; forward primer, ACACTCCAGCTGGGCCTATTCTTGGTTACT; and reverse primer, TGGTGTCGTGGAGTCG.

### Flow cytometry-based apoptosis detection, cell cycle analysis, and cell proliferation assay

For detection of apoptosis, HeLa cells (1 × 10^6^) were harvested two days after transfection, washed three times in phosphate-buffered saline (PBS), and then stained with annexin V-FITC and propidium iodide (PI), according to the manufacturer’s instructions (Beyotime Biotechnology, China). Samples were acquired via a BD FACScan^TM^ flow cytometer (Becton Dickinson, USA) and analyzed with BD FACSDiva^TM^ software 6.0 (Becton Dickinson, USA).

For the cell cycle progression assay, HeLa cells (1 × 10^6^) were harvested, washed three times with ice-cold PBS, and fixed with 70% ethanol overnight at 4 °C. The cells were stained with PI, and a cell cycle profile was determined using a BD FACSAria^TM^ III cell sorter (Becton Dickinson, USA). Ten thousand events were acquired for each sample, and the cell cycle distributions were determined using ModFit LT^TM^ software (Becton Dickinson, USA). Experiments were repeated three times, and the results were presented as the percentage of cells in a particular phase.

Thiazolyl blue tetrazolium bromide (Beyotime Biotechnology, China) was used to determine cell proliferation. Briefly, HeLa cells (1 × 10^3^) were plated in triplicate in 96-well plates. Ten microliters of cell proliferation reagents was added to each well, and the cells were incubated at 37 °C for 2 h. The cell numbers were estimated by measuring the optical density at 570 nm. Wells containing only medium without cells served as the blank.

### Protein extraction, quantification, and digestion

Harvested cells were first freeze-dried into a dry powder with a lyophilizer. The freeze-dried powder was dissolved in 200 μL of lysis buffer (7 M urea, 2 M thiourea, 4% sodium dodecyl sulfate (SDS), 40 mM Tris-HCl, pH 8.5) containing 1 mM phenylmethylsulfonyl fluoride and 2 mM EDTA. The suspension was sonicated for 15 min and then centrifuged at 4 °C and 12,000 *g* for 20 min. Then, 800 μL of cold acetone containing 10 mM 1,4-dithiothreitol (DTT) was added to the supernatant, and the mixture was incubated in a freezer overnight. After centrifugation at 12,000 rpm and 4 °C for 20 min, the precipitate was collected and washed with 800 μL of cold acetone. After centrifugation at 13,000 rpm and 15 °C for 20 min, the pellet was dried and resuspended in 8 M urea and 100 mM triethylammonium bicarbonate (TEAB; pH 8.0). Protein was quantified using the Bradford method^70^. Three biological replicates were prepared for both the wild-type and knockout lines, i.e., six samples in total. Protein samples were reduced with 10 mM DTT at 56 °C for 30 min, then alkylated with 50 mM iodoacetamide for 30 min in the dark. After diluting five times with 100 mM TEAB, an equal amount of protein from each sample was aliquoted for tryptic digestion. Trypsin was added at an enzyme–protein ratio of 1:50 (w/w), and the digestion was performed at 37 °C for 12–16 h. After digestion, peptides were desalted using a Strata-X^®^ C18 column (Phenomenex Inc., USA), and the desalted peptides were lyophilized for further analysis.

### Liquid chromatography–tandem mass spectrometry (LC-MS/MS)

LC-MS/MS analysis was performed on an AB Sciex Triple time-of-flight (TOF)^®^ 5600 + system in two phases for each sample, i.e., data-dependent acquisition (DDA) followed by SWATH acquisition, both with the same LC gradient and the same amounts of sample. For DDA, peptide samples were loaded onto a C18 trap column (particle size of 5 μm, 100 μm × 20 mm) and eluted at 300 nL/min onto a self-packed C18 analytical column (particle size of 3 μm, 75 µm × 150 mm) over a 120-min chromatography gradient (solvent A: 2% acetonitrile and 0.1% formic acid; solvent B: 90% acetonitrile and 0.1% formic acid). MS1 spectra were collected in the *m/z* range of 360–1460 for 250 ms. The 30 most intense precursors with a charge state of 2–5 were selected for fragmentation, and MS2 spectra were collected in the *m/z* range of 50–2,000 for 100 ms; precursor ions were excluded from reselection for 15 s. For SWATH data acquisition, the same chromatographic method was used as in the DDA run described above, but with a 50 ms MS1 scan followed by 32 × 25 a.m.u. isolation windows covering the mass range of 400–1,250 amu. (cycle time of 3.25 s).

### Mass spectrometry data processing and quantitation

The instrumental MS/MS data acquired under DDA mode were searched against the *Cricetidae* component of the Uniprot database using ProteinPilot^TM^ Software (v4.5, Sciex, USA). For protein identification, the ProteinPilot^TM^ Paragon algorithm was employed^71^. The parameters were set as follows: the instrument was a TripleTOF 5600; cysteines were modified with iodoacetamide; biological modifications were selected as the ID focus; and trypsin digestion. For the false discovery rate (FDR) calculation, an automatic decoy database search strategy^72^ was employed to estimate the FDR using the Proteomics System Performance Evaluation Pipeline Software, integrated in the ProteinPilot^TM^ Software. Raw data for each experimental set were searched in a single batch to create a result file that was used for subsequent analysis. The outputs of ProteinPilot^TM^, i.e., group files, were used as a reference spectral library, which contains the peptide sequence, charge state, modifications, retention time, confidence score, and the corresponding fragment ions with their *m/z* and intensity values.

Spectral library generation and SWATH data processing were performed using Skyline version 2.5 software^73^. Prior to targeted data extraction, a spectral library document was automatically generated according to the following rules: (i) peptides containing modifications and/or shared between different protein entries/isoforms were excluded; (ii) peptides with confidence less than 95% (determined by ProteinPilot^TM^) were excluded; (iii) the top five fragment ions ranked by intensities were chosen; (iv) fragment ions within the SWATH isolation window were excluded; (v) to control for the FDR, a random mass shift of Q1 and Q3 *m/z* strategy was used to create a decoy spectral library^74^. For targeted peak extraction, an *m/z* tolerance of 10 ppm was allowed for both the peptide precursor and fragment ion. Each ion’s extracted ion chromatogram (XIC) was automatically extracted within a retention time width of 5 min, and the area under the XIC curve for each individual ion was calculated. The areas of the fragment ions belonging to one peptide were summed to represent a peptide’s abundance, and a summed abundance of peptides from a given protein was used to represent the protein’s abundance. The median value of three biological replicates was used as a representative value of each group to calculate the ratio between the wild-type and the miR-26a-knockout cell lines. P < 0.05 (Student’s *t* test between the wild-type and knockout lines) and fold change >1.5 were used to determine proteins with statistically significant changes in expression. In order to eliminate random errors and sample bias, we normalized all the data across samples using a median normalization method^75^. To assess the data confidence and to control the FDR, the mProphet algorithm was employed by Skyline towards each extracted peak^76^.

### Pathway and gene ontology (GO) analyses

Pathway analysis was performed by mapping the proteins with significantly altered expression levels with pathways in the Kyoto Encylopedia of Genes and Genomes database. The miR-26a-upregulated and -downregulated proteins identified by SWATH-MS were submitted for GO analysis^77^ (http://www.geneontology.org/).

### Sequence analysis for miR-26a target prediction

To determine the frequency of miR-26a seed regions, the genes identified by the proteomics study with annotated 3ʹ-UTR were selected. The corresponding mRNA sequences were downloaded from GenBank. Pictar (http://pictar.bio.nyu.edu/), Targetscan4.1 (http://www.targetscan.org/), miRanda (http://www.microrna.org/microrna/home.do), and RNA22 (version 2; https://cm.jefferson.edu/rna22/Interactive/) and were used to computationally predict targets of miR-26a.

### Luciferase assays

The 3ʹ-UTR fragments of selected genes were amplified from human cDNA and cloned downstream of the luciferase open reading frame in pGL3-control vector (Promega, USA). Twenty four hours prior to transfection, 5 × 10^4^ cells were plated per well in a 48-well plate. pGL3 constructs (100 ng) plus 10 ng of the Renilla luciferase plasmid phRL-SV40 (Promega, USA) were co-transfected with miR-26a mimic or control oligo using Lipofectamine 2000 (Invitrogen, USA) in 293 T cells. Luciferase assays were performed using the dual luciferase reporter assay system (Promega, USA). Firefly luciferase activity was normalized to Renilla luciferase activity for each transfected well. For each experimental trial, cells were transfected in quintuplicate. For each construct, the values from the miR-26a mimic were normalized to the control oligo. A p-value was calculated with the two-tailed t-test to compare relative luciferase activities of miR-26a mimic transfection with control oligo transfection.

### Western blot

For the western blot assays, HeLa cells were first washed with PBS and then lysed in SDS lysis buffer for 5 min. The supernatant was collected as the total cell lysate after centrifugation. After protein quantitation, equal amounts of proteins were boiled, separated on a 12.5% SDS-polyacrylamide gel by electrophoresis, and then transferred to a nitrocellulose membrane. The membrane was probed with mouse monoclonal antibodies (SFN/CDK6/CDK4/CDK1/BID/BAX/CYC/ACTB, from Cusabio Biotech Co., Ltd., China; NUF2/ MYPN/DNAJC9/USP47/PPA1, from Proteintech Group, Inc., China) and horseradish peroxidase goat anti-mouse secondary antibody. Proteins were visualized using electrochemiluminescence reagents. To generate the miR-26a overexpression line, HeLa cells were plated in 12-well plates (0.5 × 10^6^ cells/well) within 1 mL medium supplemented with 10% FBS. Transfection of miRNA mimics was performed with InterferIN (Polyplus-Transfection, USA) according to the manufacturer’s instructions, followed by incubation at 37 °C for 48 hours.

### PRM

Target proteins were selected for validation by PRM analysis on a TripleTOF^®^ 5600 + LC-MS/MS system (Sciex, USA). Protein extraction and tryptic digestion were performed as described above for the SWATH experiment. The peptides monitored for each protein were selected based on the ion signal intensities in the spectral library. A list of peptides containing *m/z* and retention time information was exported from Skyline and imported into the instrumental software Analyst^®^ (version 1.7, Sciex, USA) to generate the PRM acquisition method. The PRM method was evaluated and refined to ensure reliability. Data acquisition for each sample was performed using the finalized PRM acquisition method on a quadrupole-quadrupole-time-of-flight (QqTOF) mass spectrometer (TripleTOF^®^ 5600+ system, Sciex, USA), in which each precursor ion was selected by the quadrupole, fragmented, and then all fragmented daughter ions were quantified in the TOF mass analyzer. Data processing was performed in Skyline^64^ (version 2.5), and the quantification results were manually inspected for each peptide of the targeted proteins.

## Electronic supplementary material

Supplemental Information

Dataset 1

Dataset 2

Dataset 4

Dataset 3

## Data Availability

All data generated or analyzed during this study are included in this published article (and its Supplementary Information files).
