# Supplementary material for: Label-free Quantitative Analysis of Protein Expression Alterations in miR-26a-Knockout HeLa Cells using SWATH-MS Technology
Source: Sci Rep. 2019 Feb 4;9:1399. doi: 10.1038/s41598-018-34904-8 (PMC6362012; doi:10.1038/s41598-018-34904-8)
Supplement: Supplementary file 1 — Supplemental Information [file 41598_2018_34904_MOESM1_ESM.pdf]

Label-free Quantitative Analysis of Protein Expression Alterations in miR-26a-Knockout HeLa Cells using SWATH-MS Technology

Hexiao Shen, Li Li, Zhaowei Teng, Tianqing Meng, Xiangbin Kong, Yan Hu, Yun Zhu, Lixin Ma

Supplementary information

Supplemental Figure S1. Relative comparison of miR-26a levels between the wild-type and the miR-26a knockout lines.

Supplemental Figure S2. Workflow of quantitative proteomics on the miR-26a knockout HeLa cell line using SWATH-MS technology. Three biological replicates of both wild type and miR-26a knockout lines were prepared, each containing 10^7 cells. Proteins were extracted from the harvest cells and quantified. A pooled sample of the above-mentioned six biological replicates (three for each group) were used to generate a reference library. The six individual samples were analyzed with SWATH 2.0 (Sciex, USA). All the acquired data were used for statistical analysis and relative protein quantitation across all six samples in both groups. Proteins with significant changes were further analyzed to reveal potential miR26-a functions and some were selected for validation using PRM and Western blotting.

Supplemental Figure S3. Summary of the proteomics analysis results (A) and coefficient of variations of the biological replicates (B).

Supplemental Figure S4. Pearson correlation coefficients (PCCs) of the protein abundance across the three biological replicates within each group.

Supplemental Figure S5. Full-length Western blots for (A) SFN, (B) CDK6, (C) CDK4, (D) CDK1, (E) BID, (F) BAX, (G) CYC, and (H) ACTB. Cropped images used in Fig. 5 were framed in red.

Supplemental Table 1. SWATH-MS proteomics analysis identified 3201 proteins and relatively quantified 1646 proteins from wild type and miR-26a knockout HeLa cells.

Supplemental Table 2. Proteins showing statistically significant difference (214 up-regulated and 252 down-regulated) between wild type and miR-26a knockout HeLa cells.

Supplemental Table 3. GO analysis results of the potential miR-26a target proteins.

Supplemental Table 4. Transitions of peptides used in PRM experiments for quantitation of proteins of interest.

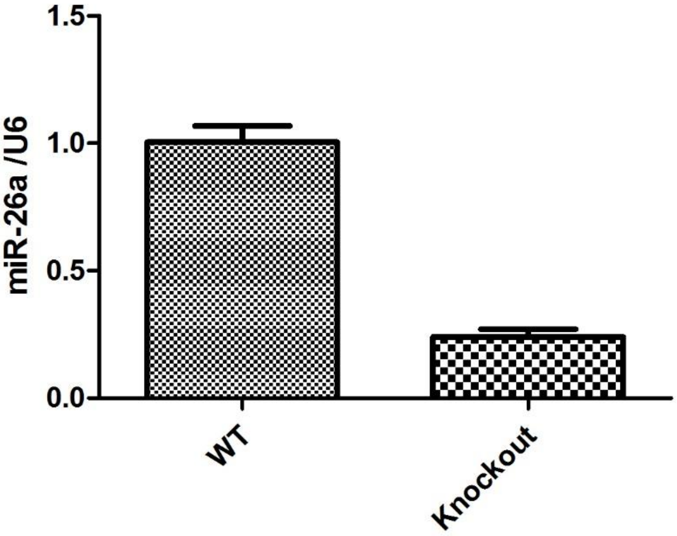

Supplemental Fig. S1

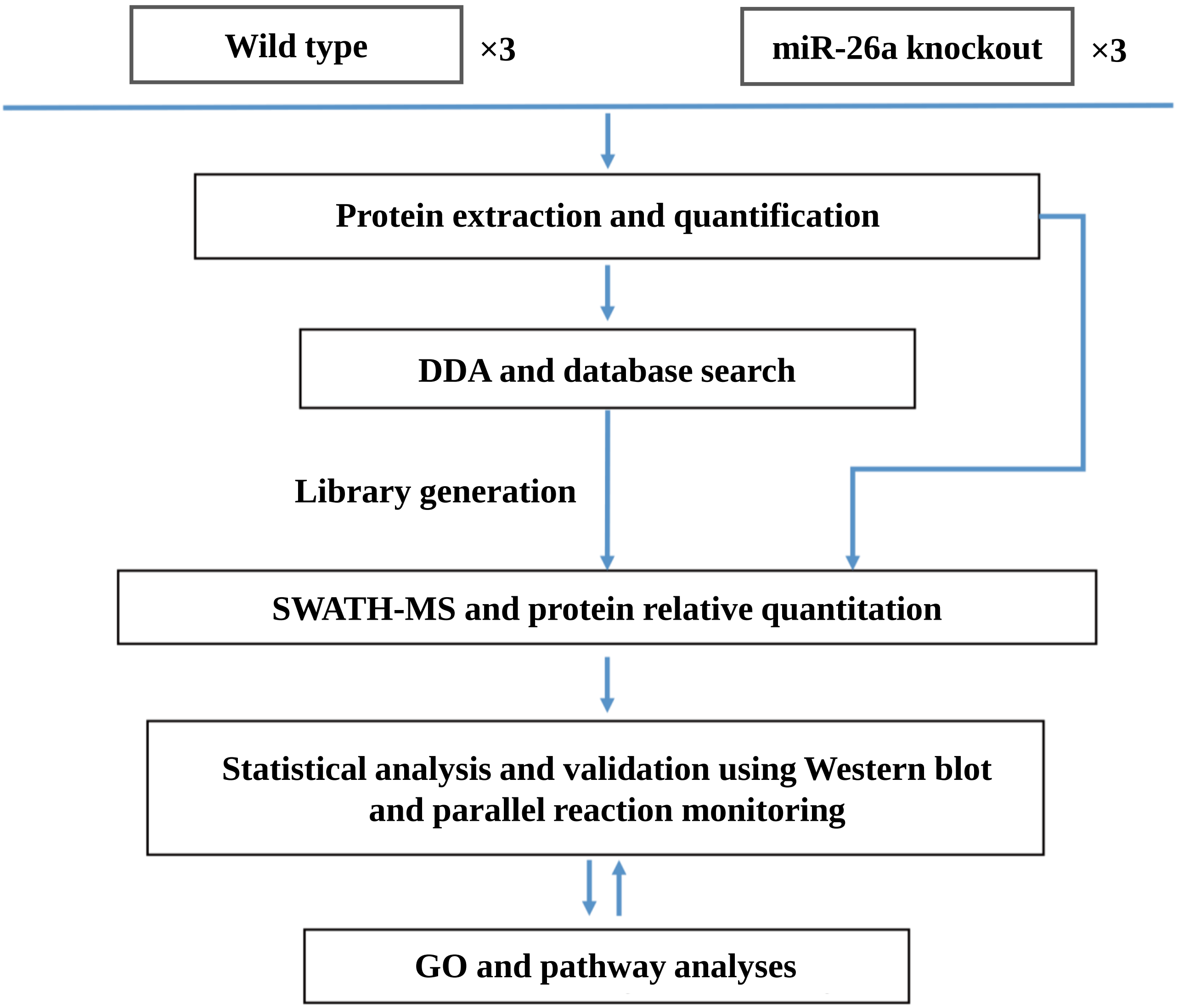

Supplemental Fig. S2

A

Type	KO/NC
**Quant Num.**	1646
**sig. diff Num.**	466
**sig. UP num.**	214
**sig. DOWN num.**	252

B

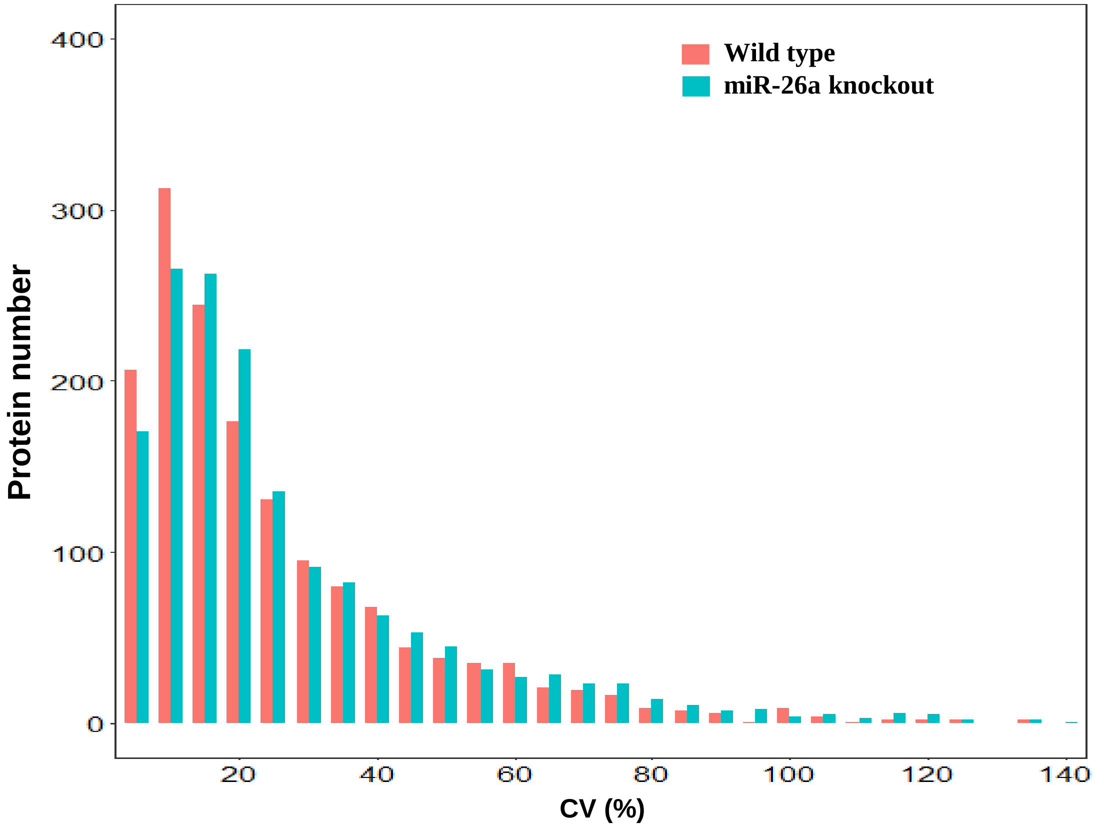

Supplemental Fig. S3

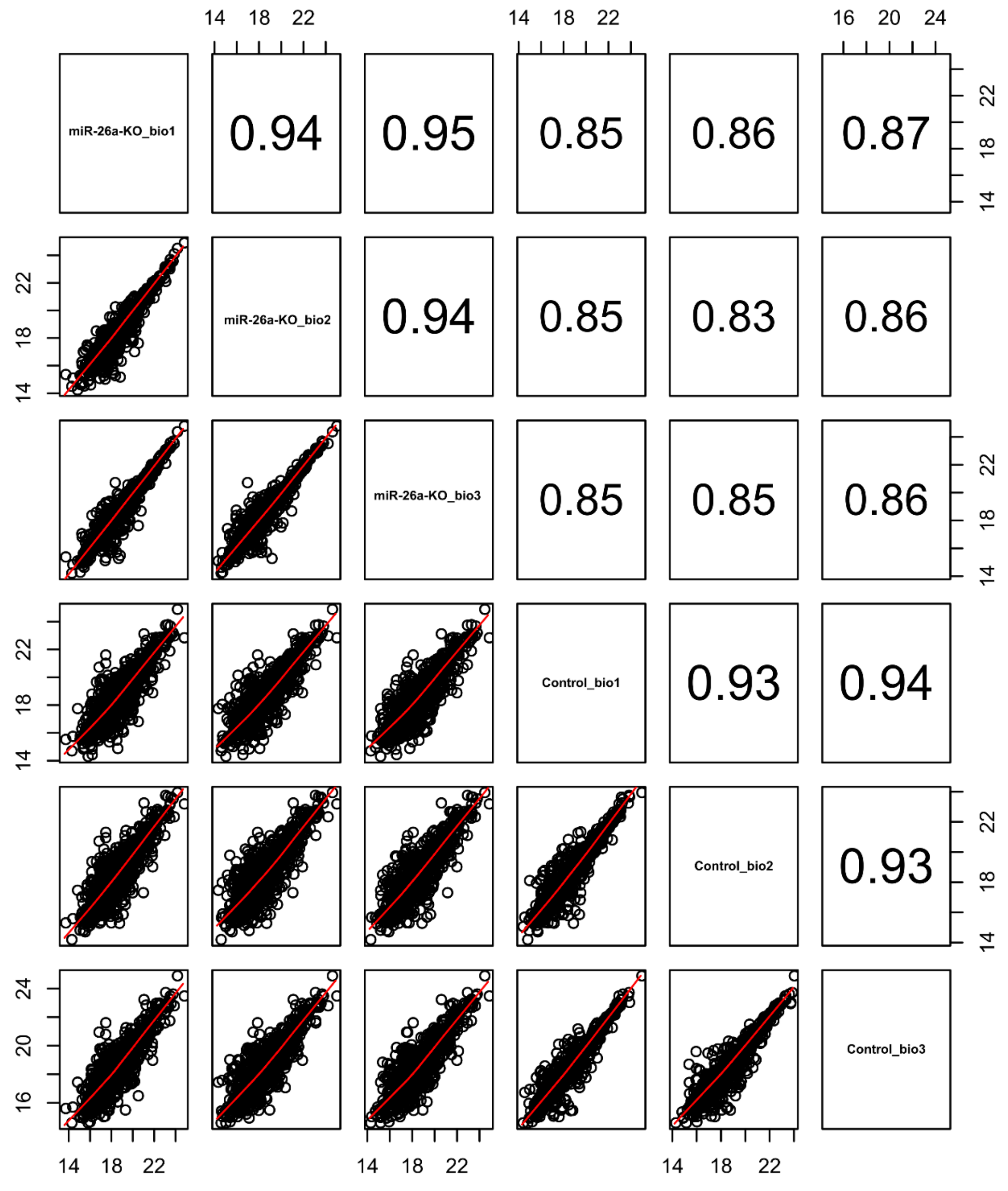

Supplemental Fig. S4

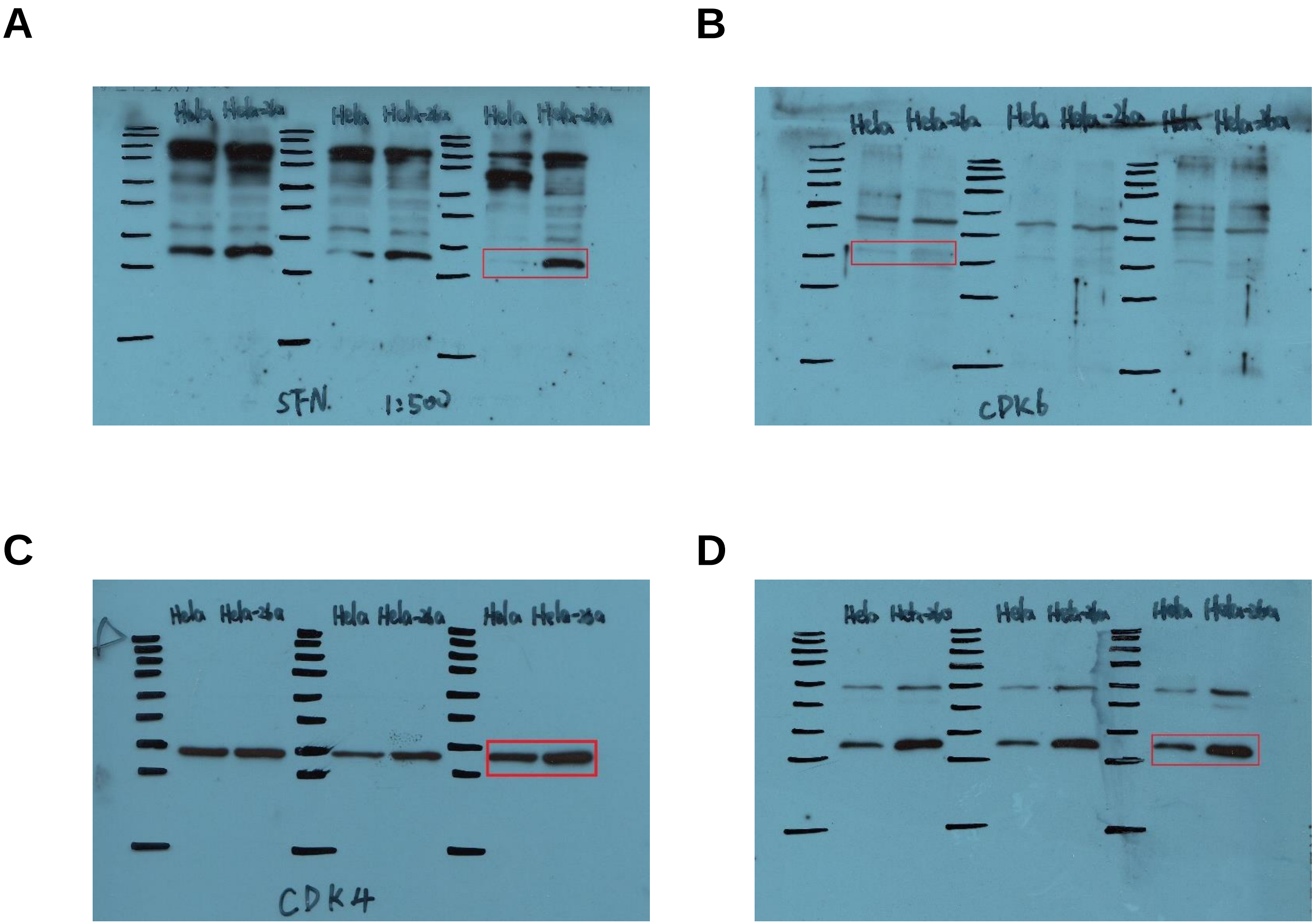

Supplemental Figure S5

E

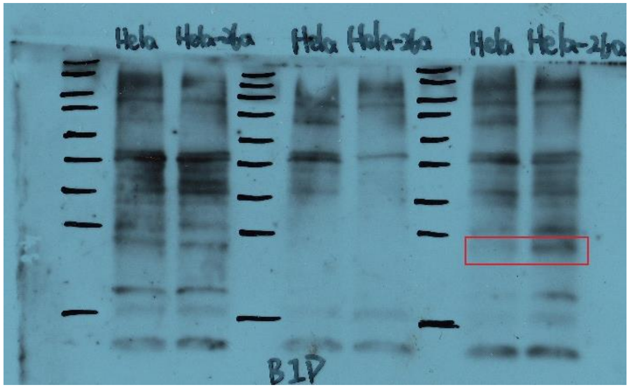

F

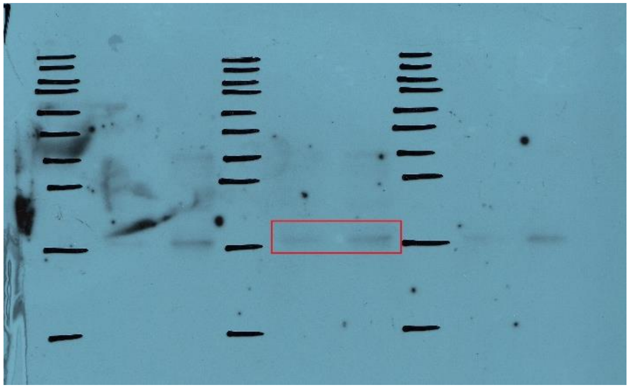

G

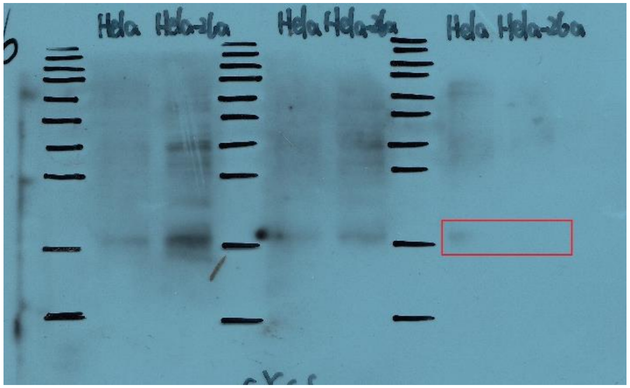

H

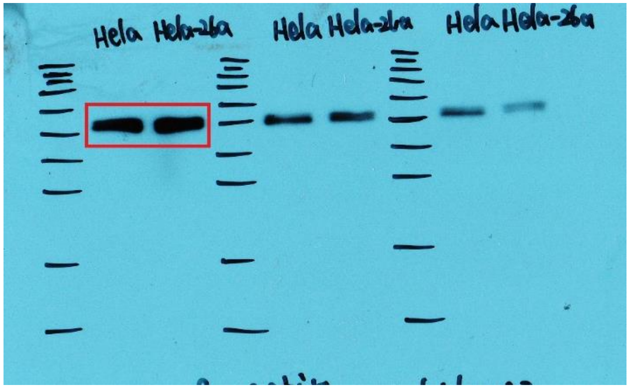

Supplemental Figure S5
